# Serum Uric Acid Is More Strongly Associated with Impaired Fasting Glucose in Women than in Men from a Community-Dwelling Population

**DOI:** 10.1371/journal.pone.0065886

**Published:** 2013-06-13

**Authors:** Ryuichi Kawamoto, Yasuharu Tabara, Katsuhiko Kohara, Tomo Kusunoki, Masanori Abe, Tetsuro Miki

**Affiliations:** 1 Department of Community Medicine, Ehime University Graduate School of Medicine, Ehime, Japan; 2 Center for Genomic Medicine, Kyoto University Graduate School of Medicine, Kyoto, Japan; 3 Department of Geriatric Medicine, Ehime University Graduate School of Medicine, Ehime Japan; University of KwaZulu-Natal, South Africa

## Abstract

Serum uric acid (SUA) levels are associated with metabolic syndrome (MetS) and its components such as glucose intolerance and type 2 diabetes. It is unknown whether there are gender-specific differences regarding the relationship between SUA levels, impaired fasting glucose (IFG) and newly detected diabetes. We recruited 1,209 men aged 60±15 (range, 19–89) years and 1,636 women aged 63±12 (range, 19–89) years during their annual health examination from a single community. We investigated the association between SUA levels and six categories according to fasting plasma glucose (FPG) level {normal fasting glucose (NFG), <100 mg/dL; high NFG-WHO, 100 to 109 mg/dL; IFG-WHO, 110 to 125 mg/dL; IFG-ADA, 100 to 125 mg/dL; newly detected diabetes, ≥126 mg/dL; known diabetes} SUA levels were more strongly associated with the different FPG categories in women compared with men. In women, the associations remained significant for IFG-WHO (OR, 1.23, 95% CI, 1.00–1.50) and newly detected diabetes (OR, 1.33, 95% CI, 1.03–1.72) following multivariate adjustment. However, in men all the associations were not significant. Thus, there was a significant interaction between gender and SUA level for newly detected diabetes (*P* = 0.005). SUA levels are associated with different categories of impaired fasting glucose in participants from community-dwelling persons, particularly in women.

## Introduction

Serum uric acid (SUA) is the final oxidation product of purine metabolism in humans. Many previous studies have shown that increased SUA levels are also associated with metabolic syndrome (MetS) and its components such as glucose intolerance and type 2 diabetes [1]. Thus, SUA seems to merely be a risk factor or marker for diabetes mellitus. However, its importance as a risk factor is still controversial. Some studies showed that there is no association between SUA level and the risk of type 2 diabetes [2], however, in a recent meta-analysis including 11 studies, SUA level was closely associated with an increased risk for incident type 2 diabetes [3], [4]. In addition, hyperinsulinemia, which is induced by insulin resistance following metabolic syndrome, causes increased SUA levels by a decrease in urinary uric acid excretion [5] and accumulation of substrates for uric acid production [6]. SUA level is significantly higher in men than in women, and its role as a risk factor is different between the genders [7]. However, most previous studies on this issue were conducted in male populations alone [4] and, if including both genders, few studies conduct gender-specific analyses [7]. Thus, it remains unknown whether there are gender-specific differences regarding the relationship between SUA level and prediabetic status.

In the present study, we investigated the association between SUA level and various categories defined by fasting plasma glucose (FPG) level in a population of 19 to 89 year-old men and women in Japan.

## Materials and Methods

### Subjects

The present study was designed as part of the Nomura study, as a longitudinal epidemiological study evaluating factors relating to cardiovascular disease (CVD), and death [8]. Subjects were selected through a community-based annual check-up process in a rural town located in Ehime prefecture, Japan. Information on medical history, present conditions, smoking, alcohol habits, and medication were obtained by individual interviews using a structured questionnaire. Smoking status was classified into non-current smoker and current smoker. Daily alcohol consumption was classified into non-current drinker and current drinker. The sample population included 1,179 men and 1,632 women because there were more women than men among medical check-up consultation in Japan. This study was approved by the ethics committee of Ehime University School of Medicine, and written informed consent was obtained from each subjects.

### Evaluation of Risk Factors

Information on demographic characteristics and risk factors was collected using the clinical files. Body mass index (BMI) was calculated by dividing weight (in kilograms) by the square of the height (in meters). We measured blood pressure with an appropriate-sized cuff on the right upper arm of the subjects in the sedentary position using an automatic oscillometric blood pressure recorder (BP-103i; Colin, Aichi, Japan) while they were seated after having rested for at least 5 min. Hypertension was defined as systolic blood pressure (SBP) ≥140 mmHg and/or diastolic blood pressure (DBP) ≥90 mmHg, or use of antihypertensive medication. A fasting venous blood sample was obtained from all study participants while sitting by the laboratory of health examination center during an overnight fast of more than 11 h. The plasma samples were immediately frozen and stored at −80°C until measurements were taken by the laboratory of our department. Plasma lipids and glucose levels were determined with an autoanalyzer (Hitachi 7250 Hitachi, Tokyo). Total cholesterol (T-C) (Ekudia L-CHO; Eiken Chemical Co., Tokyo, Japan) and Triglycerides (TG) (Auto A TG-F; MIZUHO MEDY Co., Saga, Japan), and serum creatinine analyses were carried out using the enzymatic method. High-density lipoprotein cholesterol (HDL-C) was determined by a direct method (Determina L HDL-Co; Kyowa Medex Co., Tokyo, Japan). SUA levels were measured using the uricase-perioxidase method (L-UA F; Wako Pure Chemical Industries Co., Osaka, Japan) and FPG levels were measured using the *hexokinase method* (L-Glu; Wako Pure Chemical Industries Co., Osaka, Japan). IFG was defined as a FPG level of 110 to 125 mg/dL by the World Health Organization (WHO) or 100 to 125 mg/dL by the American Diabetes Association (ADA). Known diabetes was defined as validated physician diagnosis or current use of antidiabetic medication, and newly detected diabetes was defined as a FPG level of ≥126 mg/dL. Estimated Glomerular Filtration Rate (eGFR) was calculated using the following equation; 194×Cr^−1.094^×Age^−0.287^ (×0.739 if female) [9].

### Statistical Analysis

Data are presented as the mean ± standard deviation (SD) unless otherwise specified, and for parameters with non-normal distributions (TG, FPG) the data are shown as median (interquartile range) values. In all analyses, parameters with non-normal distributions were used after log-transformation. Statistical analysis was performed using IBM SPSS Statistics Version 20 (Statistical Package for Social Science Japan, Inc., Tokyo, Japan). In these analyses, subjects were divided into six categories according to the FPG level {normal fasting glucose (NFG), <100 mg/dL; high NFG-WHO, 100 to 109 mg/dL; IFG-WHO, 110 to 125 mg/dL; IFG-ADA, 100 to 125mg/dL; newly detected diabetes, ≥126 mg/dL; known diabetes}, and differences among the groups were analyzed by student’s t-test for continuous variables or the χ^2^ -test for categorical variables. Multiple logistic regression analysis was used to evaluate the contribution of SUA for each of the six categories of impaired glucose regulation. In the analyses four models were fitted: The 1st model included SUA (continuous), age and gender (only total sample). The 2nd model included the 1st model plus, current smoker (yes/no), current drinker (yes/no), actual hypertension (yes/no), and eGFR (continuous). The 3rd model included T-C (continuous), HDL-C (continuous), lipid-lowering medication (yes/no), and urate-lowering medication (yes/no) in addition to the 2nd model, and the 4th model included BMI (continuous) in addition to the 3rd model. In a logistic regression analysis the association between SUA level and all impaired glucose regulation groups combined as the outcome was investigated. In this analysis, study participants with high NFG-WHO, IFG-WHO, IFG-ADA, newly detected diabetes, and known diabetes were regarded as persons with impaired glucose regulation, and persons with NFG were regarded as the reference group. All analyses were done for total sample as well as men and women. The interactive effect of gender and SUA for each category was evaluated using a general linear model. A *p*-value <0.05 was considered significant.

## Results

### Characteristics of Subjects

Gender-specific characteristics of the subjects categorized according to FPG levels are illustrated in Table 1 and Table 2. The study included 1,209 men aged 60±15 (range, 19–89) years and 1,636 women aged 63±12 (range, 19–89) years. Women with IFG-WHO, IFG-ADA, and newly detected diabetes had higher SUA levels than reference subjects with NFG. In both genders, FPG status categories also differed with respect to confounding factors. As compared to the NFG, men and women with High NFG-WHO, IFG-WHO, IFG-ADA, and newly detected diabetes were older, had a higher BMI as well as higher SBP, DBP and prevalence of actual hypertension. IFG-WHO, IFG-ADA, and newly detected diabetes were related to higher TG. In women with IFG-ADA, HDL-C and eGFR were lower as compared to the NFG group, and T-C was higher.

**Table 1:** Characteristics of male subjects categorized according to fasting plasma glucose.

MenFasting plasma glucose		Category				
	<100 mg/dL	100–109 mg/dL	110–125 mg/dL	100–125 mg/dL	≥126 mg/dL	Medication
	NFG	High NFG-WHO	IFG-WHO	IFG-ADA	NDD	Known diabetes
Characteristics	N = 726	N = 208	N = 136	N = 344	N = 92	N = 47
Age (years)	59±15	61±14	61±13	61±14	67±9****	68±9****
Body mass index (kg/m2)	23.0±3.0	24.2±3.1****	24.4±3.1****	24.3±3.1****	24.0±2.9*	23.4±2.6
Current smoker, %	39.5	31.2*	33.8	32.3*	21.7***	23.4*
Current drinker, %	84.2	85.6	87.5	86.3	81.5	72.3*
History of CVD, %	9.2	9.6	12.5	10.8	10.9	19.1*
Systolic blood pressure (mmHg)	135±20	145±20****	144±20****	145±20****	146±22****	148±21****
Diastolic blood pressure (mmHg)	82±11	86±11****	87±12****	87±12****	85±11	85±10
Actual hypertension, %	20.5	31.2***	36.0****	33.1****	31.5*	48.9****
Total cholesterol (mg/dL)	188±33	198±36****	190±37	195±36***	194±40	192±34
HDL cholesterol (mg/dL)	58±14	59±16	57±17	58±17	56±15	57±17
Triglycerides (mg/dL)	96 (71–138)	107 (77–154)	112 (79–183)**	108 (78–162)**	108 (84–175)*	92 (67–154)
Lipid-lowering medication, %	3.6	4.3	3.7	4.1	3.3	14.9***
eGFR (mL/min/1.73 m^2^)	80.7±17.4	81.1±17.1	81.0±18.0	81.1±17.4	80.8±18.4	78.5±19.3
Serum uric acid (mg/dL)	5.9±1.4	6.1±1.5	6.2±1.5	6.1±1.5	5.6±1.5	5.6±1.4
Urate-lowering medication, %	7.7	12.0	8.8	10.8	9.8	14.9

NFG, normal fasting glucose; IFG, impaired fasting glucose, NDD, newly detected diabetes; CVD, cardiovascular disease; HDL, high-density lipoprotein; eGFR, estimated glomerular filtration ratio.

* *P*<0.05; **P<0.01; ***P<0.005; ****P<0.001 for comparisons with NFG. Data for triglycerides was skewed, and presented as the median (interquartile range), and log-transformed for analysis.

**Table 2:** Characteristics of female subjects categorized according to fasting plasma glucose.

WomenFasting plasma glucose		Category				
	<100 mg/dL	100–109 mg/dL	110–125 mg/dL	100–125 mg/dL	≥126 mg/dL	Medication
	NFG	High NFG-WHO	IFG-WHO	IFG-ADA	NDD	Known diabetes
Characteristics	N = 1,145	N = 256	N = 106	N = 362	N = 66	N = 63
Age (years)	61±13	67±10****	68±9****	67±10****	68±10****	69±9****
Body mass index (kg/m2)	23.0±3.2	24.1±3.4****	25.0±3.5****	24.4±3.4****	25.0±4.5****	23.5±2.8
Current smoker, %	2.3	2.7	2.8	2.8	0	1.6
Current drinker, %	36.1	27.7*	28.3	27.9***	24.2	23.8
History of CVD, %	6.0	10.2*	11.3	10.5**	16.7***	7.9
Systolic blood pressure (mmHg)	135±23	144±23****	149±21****	145±22****	152±20****	143±22*
Diastolic blood pressure (mmHg)	79±12	82±12***	83±11***	82±11****	85±12****	78±9
Actual hypertension, %	21.1	39.1****	46.2****	41.2****	54.5****	44.4****
Total cholesterol (mg/dL)	207±33	215±32****	210±34	213±33***	218±30**	211±39
HDL cholesterol (mg/dL)	64±15	64±16	59±15*	62±16****	62±17	59±13
Triglycerides (mg/dL)	90 (67–125)	95 (70–139)	109 (79–149)**	97 (72–142)**	114 (86–167)****	108 (77–142)*
Lipid-lowering medication, %	5.2	9.4*	11.3*	9.9***	9.1	12.7*
eGFR (mL/min/1.73 m^2^)	80.5±17.6	77.1±16.6	76.0±16.7	76.8±16.6***	80.6±23.9	79.2±23.2
Serum uric acid (mg/dL)	4.4±1.1	4.6±1.1	4.9±1.0****	4.7±1.1****	4.9±1.3***	4.5±1.1
Urate-lowering medication, %	0.7	0	1.9	4.5	4.5*	1.6

* *P*<0.05; **P<0.01; ***P<0.005; ****P<0.001 for comparisons with NFG. Data for triglycerides was skewed, and presented as the median (interquartile range), and log-transformed for analysis.

As shown in Table 3, multiple linear regression analysis was used to evaluate the contribution of SUA for FPG status categories. In the age and gender-adjusted model the odds ratio (OR) {(95% confidence interval (CI)} was 1.12 (1.03–1.22) for high NFG-WHO, 1.22 (1.14–1.40) for IFG-WHO, and 1.17 (1.09–1.25) for IFG-ADA. Further adjustment for age, gender, current smoker, current drinker, actual hypertension, eGFR, T-C, HDL-C, urate lowering medication, and lipid-lowering medication somewhat attenuated the associations. However, the ORs per mg/dL increment of SUA remained significantly increased for high NFG-WHO (OR, 1.10; 95% CI, 1.00–1.20), IFG-WHO (OR, 1.28; 95% CI, 1.14–1.44), and IFG-ADA (OR, 1.16; 95% CI, 1.07–1.25) compared to participants with NFG. Additional adjustment for BMI, the associations between SUA level and the categories remained significant for IFG-WHO (OR, 1.21; 95% CI, 1.08–1.37) and IFG-ADA (OR, 1.10; 95% CI, 1.02–1.20). As shown in Table 3, SUA levels were more strongly associated with the different FPG categories in women compared with men. In women, after multivariate adjustment, the associations remained significant for IFG-WHO (OR, 1.23, 95% CI, 1.00–1.50) and newly detected diabetes (OR, 1.33, 95% CI, 1.03–1.72), but the significance was attenuated for IFG-ADA. However, in men, the multivariate analyses showed that all associations were not significant. Thus, there was a significant interaction between gender and SUA level for newly detected diabetes (*P* = 0.005).

**Table 3:** Association of serum uric acid levels with IFG-WHO, IFG-ADA, newly detected diabetes, and known diabetes compared with normal fasting glucose.

CharacteristicsFasting plasma glucose	Category					
	<100 mg/dL	100–109 mg/dL	110–125 mg/dL	100–125 mg/dL	≥126 mg/dL	Medication
	NFG	High NFG-WHO	IFG-WHO	IFG-ADA	Newly detected diabetes	Known diabetes
Men	N = 726	N = 208	N = 136	N = 344	N = 92	N = 47
Women	N = 1,145	N = 256	N = 106	N = 362	N = 66	N = 63
Model 1 Total	Reference	**1.12 (1.03–1.22)**	**1.22 (1.14–1.40)**	**1.17 (1.09–1.25)**	1.06 (0.93–1.20)	0.97 (0.83–1.13)
Men	Reference	1.09 (0.98–1.22)	**1.15 (1.01–1.32)**	**1.12 (1.02–1.23)**	0.91 (0.78–1.07)	0.93 (0.75–1.15)
Women	Reference	1.10 (0.97–1.24)	**1.35 (1.14–1.60)**	**1.17 (1.05–1.30)**	**1.36 (1.11–1.68)**	1.00 (0.79–1.26)
Model 2 Total	Reference	**1.13 (1.04–1.24)**	**1.28 (1.15–1.43)**	**1.18 (1.10–1.28)**	1.14 (0.99–1.31)	1.03 (0.87–1.22)
Men	Reference	1.10 (0.97–1.24)	**1.16 (1.01–1.34)**	**1.12 (1.01–1.24)**	0.96 (0.81–1.13)	0.96 (0.81–1.13)
Women	Reference	1.10 (0.96–1.26)	**1.40 (1.16–1.69)**	**1.19 (1.05–1.33)**	**1.60 (1.26–2.02)**	1.09 (0.85–1.41)
Model 3 Total	Reference	**1.10 (1.00–1.20)**	**1.28 (1.14–1.44)**	**1.16 (1.07–1.25)**	1.08 (0.94–1.25)	0.96 (0.81–1.14)
Men	Reference	1.05 (0.93–1.19)	**1.17 (1.01–1.36)**	1.10 (0.98–1.22)	0.92 (0.78–1.10)	0.87 (0.69–1.11)
Women	Reference	1.09 (0.95–1.26)	**1.35 (1.11–1.64)**	**1.17 (1.03–1.32)**	**1.47 (1.14–1.88)**	1.02 (0.78–1.33)
Model 4 Total	Reference	1.05 (0.96–1.15)	**1.21 (1.08–1.37)**	**1.10 (1.02–1.20)**	1.03 (0.89–1.19)	0.95 (0.79–1.13)
Men	Reference	1.03 (0.91–1.17)	1.15 (0.99–1.34)	1.08 (0.97–1.20)	0.90 (0.76–1.08)	0.87 (0.68–1.11)
Women	Reference	1.02 (0.88–1.18)	1.23 (1.00–1.50)	1.07 (0.95–1.22)	1.33 (1.03–1.72)	0.99 (0.75–1.30)
P-value for serum uric acid * sex interaction		0.737	0.349	0.940	0.005	0.520

Odds ratio expressed per mg/dL increment in serum uric acid value; if the confidence intervals around the odds ratio overlap, then the odds ratios are not significantly different. Model 1: adjusted for age and gender (only total sample). Model 2: adjusted for age, gender, current smoker, current drinker, actual hypertension, and eGFR. Model 3: adjusted for age, gender, current smoker, current drinker, actual hypertension, eGFR, total cholesterol, HDL-cholesterol, lipid-lowering medication, and urate-lowering medication. Model 4: adjusted for age, gender, current smoker, current drinker, actual hypertension, eGFR, total cholesterol, HDL cholesterol, lipid-lowering medication, urate-lowering medication, and body mass index. Data for triglycerides was skewed and log-transformed for analysis.

## Discussion

In 2,845 community-dwelling persons, we determined that SUA levels are associated with different categories of impaired fasting glucose, independent of known metabolic confounding factors. SUA levels were more strongly associated with the different FPG categories in women compared with men. In women after multivariate adjustment the associations remained significant for IFG-WHO and newly detected diabetes, but in men these associations were not significant. Interestingly, we found that there was a significant interaction between gender and SUA level for newly detected diabetes.

Prior studies have reported that increased SUA level is correlated with lifestyle factors (e.g., alcohol consumption) and various metabolic factors (e.g., hypertension, hypertriglyceridemia, and low HDL-cholesterolemia) which are components of metabolic syndrome. Thus, it is possible to establish whether the observed positive association between SUA level and IFG category is noncausal. In the present study by multiple logistic regression analysis, we could demonstrate that increased SUA level among women was associated with each single category of IFG. However, this association was attenuated in men. These findings support a possible role of uric acid in the early pathogenesis of type 2 diabetes, especially in women. Hyperuricemia had a high specificity for women with IFG and newly detected diabetes, which are strongly associated with insulin resistance.

We cannot explain the underlying mechanism that accounts for the gender difference from this study. A partial explanation for this result could be alcohol consumption, which is more likely to be higher in men, the use of antihypertensive medications such as diuretics, which are known to increase SUA levels [10], and the influence of sex hormones [11]. We however, demonstrated that this result remained significant after adjustment for current drinker and actual hypertension, suggesting that drinking status and actual hypertension does not dramatically affect it. Moreover, Kolz et al. found that the minor allele for rs734553 in SLC2A9 has greater influence in lowering SUA levels in women and the minor allele of rs2231142 in ABCG2 increases SUA levels more strongly in men compared to women [12].There are strong gender-specific effects in the genetic basis of uric acid production, possibly suggesting a genetic basis for the gender differences even in glucose metabolism [13]. Thus, we thought that gender-specific analyses were also required because at all ages, the SUA level is higher in men than in women [14].

The mechanisms by which UA can have an effect on glucose metabolism are not completely understood. In the past, most authorities have viewed UA as biologically inert or possibly anti-inflammatory because of its function as an antioxidant [15]. However, recent studies have demonstrated that increased SUA may also reflect systemic inflammation [16] and oxidative stress [17] both of which are closely associated with the pathogenesis of type 2 diabetes [18]. SUA reduction of the levels of endothelial nitric oxide (NO), which is a mediator of insulin action and increases blood flow to skeletal muscle, induces endothelial dysfunction and enhances insulin resistance in the liver, muscle, and adipose tissues [19]. In addition, SUA induces activation of the renin angiotensin system [20]. The combined effects of insulin resistance and increased SUA levels may induce IFG and the development of diabetes [7].

There are some limitations to this study. First, our cross-sectional study design does not eliminate potential causal relationships between SUA and IFG. Second, the prevalence rate of the six categories of impaired glucose regulation is based on a single assessment of blood, which may introduce a misclassification bias. Third, we could not eliminate possible effects of the underlying diseases, and medication, especially diuretic use on the results. These points need to be addressed again in a large population-based sample in a prospective manner.

### Conclusions

The present study showed that the SUA levels is significantly associated with different categories of IFG, independent of other confounding factors in community-dwelling persons, particularly in women, and there is a significant interaction between gender and SUA level for newly detected diabetes. The underlying mechanism behind this relationship is unclear, but seems to be independent of traditional cardiovascular risk factors such as age, BMI, current smoking status, current alcohol consumption, active hypertension, renal function, lipids, and use of urate-lowering medication. For community-dwelling healthy persons, prospective population-based studies are needed to investigate the mechanisms underlying this association to determine whether intervention, such as effective lifestyle modifications or medication that decrease SUA in adults, will decrease the risk of increased FPG.
